# Centering community voices: advancing health equity for people and pets in Los Angeles County through community-based participatory research

**DOI:** 10.3389/fvets.2025.1539811

**Published:** 2025-06-18

**Authors:** Celeste Morales, Miguel Ruelas, Terryl Daluz, Elizabeth Analco, Nicole Vera, Janet Rivera, Megan Covington, Sloane M. Hawes

**Affiliations:** ^1^Research and Development Companions and Animals for Reform and Equity, Baltimore, MD, United States; ^2^Loving Paws, Inc., Los Angeles, CA, United States; ^3^Communities for a Better Environment, Los Angeles, CA, United States; ^4^Independent Researcher, Los Angeles, CA, United States

**Keywords:** pets, health equity, housing, environmental justice, trauma-informed care

## Abstract

**Background:**

Pets are increasingly seen as members of the family unit in U.S. households. To advance health equity and improve health service providers’ understanding of how to best support pet owners, this study aimed to understand the priorities and barriers to human and companion animal (pet) well-being services and resources in Los Angeles County, CA, USA.

**Methods:**

A community-based participatory research (CBPR) approach was used to conduct five semi-structured focus groups in May 2024 with 27 pet owners in Los Angeles County, CA, USA. Data were analyzed using an inductive approach.

**Results:**

Several themes for improving health services for people and pets were identified, including: understanding community-specific priorities for people and pets in Los Angeles County; addressing barriers to services and information for people and pets; addressing the need for affordable pet inclusive housing and tenants rights; and addressing the need for improved access to pet inclusive green spaces and environmental justice. The participants shared about the importance of mutual aid and collective care when faced with a lack of access to services.

**Discussion:**

These findings can be used across health services to inform the development of equitable, accessible, and community-specific solutions that improve the quality of life for both people and their pets in Los Angeles County, CA, USA.

## 1 Introduction

In the United States, it is estimated that 66% of households (86.9 million homes) have a companion animal (pet), and 97% of pet owners consider their pets to be a part of their family (1, 2). Several studies have demonstrated the positive impacts of pet ownership and the human-animal bond (e.g. (3–5),). However, several scholars have argued that the current definitions of “responsible pet ownership” in the U.S. are rooted in racism and perpetuate health inequities for racialized communities and their pets (6, 7). The reason for this can be, in part, attributed to the lack of diversity within the animal welfare and protection industry (e.g., veterinarians, animal control officers, behaviorists, animal shelter professionals), with the majority identifying as white women (85%) (8). This absence of racial diversity among practitioners in the field leads to significant barriers to Black, Indigenous, and People of Color (BIPOC) workforce development and a critical lack of culturally responsive information regarding pet well-being and preventative care practices (9–11). There are also pervasive systemic issues in the animal welfare and protection industry that disproportionately impact BIPOC communities and their pet family members, including but not limited to over-policing and a lack of access to food, housing, pet enrichment, behavior support, and veterinary care (11–17). To this end, in recent years, scholar-activists have highlighted the critical need to “expand the narrow notion of pet well-being to include critical justice issues like gender and sexual diversity, racial equity, economic and housing security, disability rights, and environmentalism” (18). To do so, requires a focused effort to engage in interdisciplinary collaboration with health services professionals and other disciplines such as public health, legal services, and social work.

As articulated by Khan, Iwai, and DasGupta (19): “We can choose to continue practices that perpetuate structural racism, or we can dismantle them and rebuild more just systems of care. The question then becomes: What tools and models do [we] have at our disposal to accomplish the latter goal?” While the path to health equity for people and pets will likely vary based on lived experience and historic community context, a number of tools and approaches are used in the public health field that can be leveraged in the animal welfare and protection field. One crucial tool is community-based participatory research (CBPR), which can be used to co-create effective and sustainable policies and programs grounded in understanding community-specific challenges and community-led solutions (20). Fleming et al. (21) describe CBPR as an approach that takes research and evaluation efforts “beyond simply documenting inequities and instead actively transforms inequitable systems.” CBPR involves equitable partnerships with community-based organizations and individuals throughout a research or evaluation process. This approach is particularly effective due to community partners’ knowledge of local contexts and their relationships with policymakers and the fact that they are uniquely positioned to identify problems, engage community members in research and interventions, disseminate research findings, and mobilize community members and organizations to advocate for change,” (22).

The purpose of this study was to use a CBPR approach to identify community-specific needs and priorities around human and pet well-being services in Los Angeles County, California, U.S. The data collected through this study can be used by health service providers to co-create equitable, accessible, and community-specific policies and programs that will transform the human and pet well-being service systems in Los Angeles County, CA and improve the quality of life for both people and their pets. This paper presents the findings from the focus groups that were conducted as the initial data collection phase of the project.

## 2 Materials and methods

### 2.1 The approach: community-based participatory research

This study was grounded in the evidence-based approach of community-based participatory research (CBPR). CBPR is “a partnership approach to research that equitably involves community members, practitioners, and academic researchers in all aspects of the process, enabling all partners to contribute their expertise and share responsibility and ownership.” (20). CBPR offers an opportunity to break down the typical power imbalances that often exist in traditional research practices, including a long history of treating community members as “subjects rather than co-creators of knowledge,” (23). In contrast, the CBPR approach “centers community members’ expertise through their lived experience” and requires researchers to embody a deep commitment to “forming long-term mutual relationships” with the community partners (23).

In Chicago Beyond’s (24) guidebook for using a CBPR approach, titled ‘Why Am I Always Being Researched?’, the authors reference Bryan Stevenson, founder of the Equal Justice Initiative, who explains:

*…“Getting proximate” changes our capacity to make a difference. Traditional research does not have norms for this. One starting point is face-to-face engagement between researchers and community, participants, staff, and partners. While community-based research practices offer examples of how to structure this engagement, this is human-to-human work, not a check-the-box exercise to create a particular community hearing or steer committee structure. Spending time with the organization and breaking bread fosters relationships and understanding that matter at a human level, and equips researchers to recognize—and as a result address—ways the power dynamic gets in the way of impact (p. 68).*

Research partners were recruited for this study based on the criteria of being a human and/or pet service organization that provides services in Los Angeles County, USA. The research team reached out to potential partners and provided a written project overview document. They then met by Zoom video conference to discuss the project and address any questions. Once an organization confirmed their interest in participating as a research partner, the research partner organization completed a Memorandum of Understanding (MOU), which outlined both the research partner organization and the research team’s commitments and responsibilities. These commitments included supporting recruitment for the study and attending quarterly meetings throughout the course of the project. Five Los Angeles County-based organizations participated in the project as research partners: Communities for a Better Environment, Downtown Dog Rescue, Housing Equity Advocacy and Resource Team (HEART) LA, Loving Paws, and The Paw Mission. Stipends of $500 USD were provided to each research partner as compensation for their involvement.

Training was provided to all research partners on how to recruit focus group participants. All recruitment materials were provided by the research team. The research partners were also invited (not required) to be focus group facilitators, and those individuals who facilitated received additional hourly compensation. Additional training for the focus group facilitators was provided by the research team. Throughout the project, research partners were asked to provide input and feedback on the focus group questions, any changes related to the data collection plan and timeline, and data dissemination activities. The process for getting input and feedback included research partners providing feedback directly in documents and/or discussing pertinent topics through email or a virtual meeting.

### 2.2 Characteristics and positionality of the research team

The research team’s intersectional identities undeniably shaped this study by influencing how the research was designed, implemented, and interpreted (25). Our identities also influenced how participants perceived the research and the degree of trust needed to respond to questions related to the topic of health equity for people and their pets. The identities and lived experiences of the research team had some notable similarities and differences from the research participants. Most of the authors are BIPOC individuals, with one author who is white. The educational background of the authors varies from high school degree to PhD degree. The majority of the authors are American citizens, with one author who is a Canadian citizen. Five of the authors are LA residents. Most of the authors are renters, with two authors being homeowners. All authors are pet owners. Some of the authors have personally experienced struggling to find affordable rental housing that allowed their pets. Some authors have personally experienced housing insecurity, eviction, foreclosure, homelessness, housing discrimination, and pet breed discrimination. The authors’ professional experiences include: tenants’ rights and affordable housing, housing authority leadership, social work, political science, sociology, practice experience with the community cultural wealth model, academic research and administration, research and evaluation, dog grooming, dog training, and animal sheltering. These various professional roles and experiences have resulted in a robust network of existing relationships with proximate leaders and community members in the focus community for this study. These existing relationships were critical for recruiting and engaging the individuals with lived experiences who participated in the research.

### 2.3 Data collection

Focus groups are used often in CBPR due to their conversational nature, which allows participants to share their personal experiences and perspectives in a way that “teases out nuances and tensions of complex topics and subjects,” (26). As such, this data collection method was chosen as a starting point to gain insight from Los Angeles County pet owners on the research questions.

#### 2.3.1 Ethics approval

As a coalition of nonprofit organizations rather than academic institutions with access to an Institutional Review Board, formal ethics approval and oversight were not obtained. However, the research team has completed human subjects research training (CITI Program) and the project was designed to ensure the data collection procedures met the criteria for exemption, including: minimal risks to study participants, confidentiality of participants and any identifying information, and there was no use of deception. Written informed consent was obtained from all subjects involved in the study. Signed consent forms, interview audio files, and focus group transcripts were all stored in a password secured data storage system hosted by Companions and Animals for Reform and Equity (GoogleDrive).

#### 2.3.2 Recruitment process

There were three inclusion criteria for participation in the focus groups. Participants had to be a current resident of Los Angeles County, 18 years of age or older, and they had to be either current, past, or future pet owners. The category of ‘future pet owners’ was included based on the understanding that some individuals may want to have a pet, but have been unable to due to the systemic barriers that make pet ownership inaccessible for many. These inclusion criteria were selected to ensure the broadest possible representation of Los Angeles County pet owners.

Participants were recruited through word of mouth and paper flyers distributed by the five research partner organizations. The research partners invited potential participants to fill out a form with their schedule preferences and availability. They were then contacted by the project lead to confirm their participation in the focus group.

To minimize barriers to participating in the focus groups, recruitment materials were provided in both English and Spanish, the focus groups were held in two different geographic locations of Los Angeles County (Van Nuys, CA and Southgate, CA), childcare was provided, food was provided, and participants were able to select their language preference for participation (English, Spanish, or bilingual). Multiple date and time options were listed in the sign-up form for participants to select from.

#### 2.3.3 Instrument development

The focus group questions were co-created by the research team and the research partner organizations through a collaborative process that began with an in-person meeting to discuss the vision and goals for the project. At the meeting, research partners were asked questions about their organizations’ priorities and impact areas, their ideal study outcomes for both their organizations and the communities they work with, and the topics and issues that they would like to explore. Project design components and preferred communication norms were also discussed. Following the meeting, a draft of the focus group questions was created based on the discussion with the research partners. This draft was then shared with the research partners, who reviewed and provided feedback on the focus group questions before they were finalized. The focus group questions can be found in Appendix 1.

#### 2.3.4 Focus group procedures

Five focus groups were held in-person in groups of 3–7 participants and were 1.5–2 h in duration. The semi-structured focus groups had a total of 18 questions. Focus groups were held in either English or Spanish. The focus groups each began with the written consent process and two icebreaker questions. Participants were then guided through the questions by two focus group facilitators. A third member of the research team attended through video conference to take notes. All participants received a $150 Visa gift card incentive at the conclusion of the focus group. The incentive amount was selected based on the recommendation of the National Health Council’s Patient Compensation Fair Market Value calculator (27).

#### 2.3.5 Sample

Twenty-seven individuals living in Los Angeles County, CA participated in the focus groups between May 1 and 7, 2024. Participant ages ranged from 18 to 74 years, with an average age of 45 years. Twenty-one (81%) of the participants identified as Hispanic/Latino/a, 4 (15%) identified as Black/African American, 1 (4%) identified as Native American/American Indian/Indigenous, and 1 (4%) identified as White, non-Hispanic. Of the twenty-seven participants, 24 (89%) were current pet owners, 5 (19%) were past pet owners, and 4 (15%) planned to own pets in the future. Because participants could select more than one answer to the demographic questions, these totals may add up to greater than 100%. Additional demographic information about the participants can be found in Table 1.

**Table 1:** Focus group participant demographics.

		Frequency	Percentage
Ages of household members^*^	Older Adult (60 + years old)	8	29.63%
	Adult (25 + years old)	21	77.78%
	Young Adult (18–24 years old)	8	29.63%
	High School (14–18 years old)	8	29.63%
	Middle School (12–13 years old)	5	18.52%
	Middle Childhood (6–11 years old)	8	29.63%
	Early Childhood (2–5 years old)	3	11.11%
	Infant and Toddlers (0–2 years old)	2	7.41%
Current housing status	Temporary Housing (living with family/friends, hotel/motel)	3	11.11%
	Renter (and my unit does not allow pets)	3	11.11%
	Renter (and my unit allows pets)	18	66.67%
	Renter (and my unit allows certain pets)	2	7.41%
	Prefer not to answer	1	3.70%
Primary form of transportation	Personal Car	14	51.85%
	Public Transit—Subway	5	18.52%
	Public Transit—Bus	8	29.63%
	Shared Car	1	3.70%
	Uber/Lyft	1	3.70%
	Other	1	3.70%
Languages spoken/written/read	English	8	29.63%
	Spanish	8	29.63%
	Spanish and English	10	37.04%
Race/ethnicity^*^	Black/African American	4	14.81%
	Hispanic/Latino/a	21	77.78%
	Native American/American Indian/Indigenous	1	3.70%
	White, non-Hispanic	1	3.70%
Level of education	Have not completed high school	3	11.11%
	Completed high school/no college	8	29.63%
	Some college/associate’s degree	13	48.15%
	Bachelor’s degree	1	3.70%
	Prefer not to answer	1	3.70%
How pets were acquired^*^	Neighbor, Friend, or Family Member who breeds animals	6	22.22%
	Neighbor, Friend or Family Member who does not breed animals (rehomed)	5	18.52%
	Other breeder	1	3.70%
	Online (Craigslist, Facebook, etc.)	1	3.70%
	Pet Store	1	3.70%
	Shelter, Humane Society, or Rescue	6	22.22%
	Other	9	33.33%
	Prefer not to answer	2	7.41%
Pet ownership status^*^	I currently have pet(s).	24	88.89%
	I have had pet(s) in the past.	5	18.52%
	I plan to have pet(s) in the future.	3	11.11%

* The total percentage exceeds 100% because participants could select more than one option for this question.

### 2.4 Data processing

Audio and video of each focus group was recorded and transcribed using Zoom (Zoom Video Communications, Inc.). The transcript files were downloaded and labeled with the date and time of the focus groups. The transcripts were then stored in a password protected database (Google Drive) hosted by one of the research partner organizations, Companions and Animals for Reform and Equity (CARE). To ensure participant confidentiality, the transcripts were reviewed line by line by one member of the research team to remove any participant identifying information. Each participant was assigned a numerical identifier. Any statements made by that participant or reference made to that participant was replaced with this numerical identifier in the transcript. The participants are referred to using these numerical identifiers in the summary of findings below. As a final stage of processing, a member of the research team listened to the audio file from each focus group and compared it with the written transcript, making any corrections needed to ensure an accurate transcript.

### 2.5 Data analysis

Analysis was conducted using the Atlas.Ti qualitative analysis software (Lumivero, LLC). Two members of the research team engaged in the analysis process using an inductive approach (25, 28). The researchers reviewed each of the transcripts line by line and generated an initial list of codes based on the concepts or phrases shared by the focus group participants. *In vivo* words or phrases from participants (the exact words or phrases used by research participants to capture their experiences or perspective) were used in this initial stage of coding to minimize researcher bias in the analysis process. The researchers then grouped together the codes into themes and subthemes. Both researchers met regularly to engage in reflexivity (25).

In alignment with best practice for cross-language qualitative research, the data were maintained in the source language they were gathered for the coding and theme development stages of the analysis process (29, 30). Further, the data analysis team included two bilingual (English and Spanish) and multicultural (American, Salvadoran, Canadian) team members with qualitative research experience (31). The focus groups conducted in English were coded and grouped into themes by one bilingual research team member, while the focus groups conducted in Spanish were coded and grouped into themes by a different bilingual research team member. The researchers then combined the English and Spanish themes, where appropriate, while maintaining separate themes for distinct concepts in English or Spanish. The research team then created written summaries of the thematic findings.

#### 2.5.1 Trustworthiness and member checking

Theoretically, the themes produced by the inductive coding procedure and use of *in vivo* codes allows for the insights from the participants to remain intact and authentic to their individual experiences. However, to minimize the extent to which data analysis is biased by the research team’s previous experiences or positionalities, the final step of the qualitative analysis process included systematically scrutinizing analysis conducted in the previous stages to verify that the clustered themes were representative. Member checking was completed where the thematic findings were shared with the research participants by email or text (depending on their contact preference) and they were invited to share feedback to ensure that their words and thoughts were represented accurately.

## 3 Results

Several themes related to needs and priorities for advancing health equity for people and pets were identified from the analysis of the focus group findings. These themes are described in detail below.

### 3.1 The importance of pets

All participants, regardless of being current or previous pet owners, viewed their pets as having positive impacts on their health and overall well-being. These positive impacts included: mental health support; love and unconditional love; emotional support and strength; safety, security, and protection; comfort; happiness; help with loneliness; companionship; responsibility; important during difficult times; and positive impacts on the community. Participants shared about the impact of their pets on their well-being, sharing sentiments such as “They made our life so much better,” (Participant 2) and “She saved me,” (Participant 3).

Many participants shared that they see their pets as their best friends, family, and children. Participant 18 shared: “…they were just my safe space, my comfort zone, because they made me feel like regardless of whether I had family or not…they were my family. And so they made me feel safe, and they showed me what actual love feels like when I did not really feel that sense of love.”

Many participants also expressed the belief that pet ownership is a fundamental right regardless of financial or housing status. Participant 1 expressed that people deserve pets “regardless of income” and “regardless of where you live,” while Participant 6 explained that everyone has the fundamental right to have dogs, as long as they are able to care for them (“Todos tenemos derecho al gran interés fundamental de tener perros siempre y cuando se tengan las actitudes de poder cuidar al animalito”). Further, Participant 2 explained that many people take care of their pets even when navigating precarious life circumstances. This participant explained: “My brother was homeless for years. He was an alcoholic, and he had a dog the whole time, and that dog looked pristine.” Participants 1, 2, and 3 all agreed that homelessness is not an issue when it comes to taking care of a pet. However, multiple participants noted that people should not be allowed to have pets if they are abusive to them, do not treat them well, and/or use the dogs for negative purposes. Participants 23 and 27 had a somewhat different perspective and noted that while they believe pet ownership is a fundamental right, people should not get a pet if they cannot afford to care for them.

### 3.2 Community-specific priorities

Participants identified several community-specific priority areas related to human and pet well-being in Los Angeles County, CA, USA. They also shared their vision for a healthy and equitable community. Participants identified the following as priorities:

- Food stamps being inclusive of pet care and supplies;
- Affordable housing including lower rent costs and pet deposits or alternatives to pet deposits;
- Financial support for pet-related housing deposits;
- Low-cost veterinary care including preventative care, vaccines, check-ups, wellness, spay and neuter, monthly membership for low-cost pet services;
- Affordable pet care services including low-cost grooming, behavior training and enrichment, dog walking services, pet ownership classes, and pet insurance;
- Opportunities to learn about responsible pet ownership including understanding nutrition, safety, exercise, medical care, emotional/behavioral needs and management, and pet waste disposal;
- More services and supplies that are available for unhoused people and their pets;
- Clean/comfortable environments including the regular maintenance of parks, dog waste bag stations in parks, water, benches, and more trash cans in public spaces and on sidewalks;
- Safer communities, including the need for dedicated pet and child areas (both indoor and outdoor) because of safety concerns;
- Walkable access to green spaces that have play areas for pets and children;
- Off-leash dog parks in close proximity (walking distance);
- Community members being included in the planning, development, and upkeep of green spaces to foster a sense of ownership and responsibility;
- Consistency and reliability from organizations, including offering regularly scheduled services on specific days;
- Mobile or pop-up health clinics for humans and pets;
- More information sharing from organizations and a centralized place for information;
- Support with service dog certification and emotional support animal letters;
- More pet stores, grocery stores/markets, and doctor’s offices
- Ethical and safe pet rescue organizations that will not “steal” pets or use dangerous and unethical methods that compromise pet health;
- Enforcement for people who abuse pets;
- Respect between neighbors regarding what each chooses to do with their space.

### 3.3 Barriers to services and information

Participants shared about their experiences with accessing human and pet well-being services in Los Angeles County. The general agreement among participants was that more services are needed to support human and pet well-being in Los Angeles County. Some participants did not know of any existing services, while others noted that many services had been impacted by COVID-19.

The focus group participants discussed several barriers and challenges to obtaining human and pet well-being services and resources in Los Angeles County. Participants shared difficult stories about the impacts of the lack of access to services, including the experience of being separated from their pets due to a lack of access to resources.

#### 3.3.1 Lack of information

One significant challenge participants identified was a lack of information. Several participants shared that they are unsure how to access information about what services are available and where or when to access them, because providers do not advertise and share information about their services in an accessible way. Participant 21 explained: “If something is inaccessible because there’s no information, that’s like a legit problem… People should be, or at least have the knowledge of ‘Oh, if I did need this, this is the place I go to.’ And then that is likely to inform other people.” Participant 1 suggested that social services should do a better job of informing people about what services clients can access. For example, Participant 1 explained “I’m on EBT and I did not even know that you could get your animal spayed or neutered by being on EBT…not even somebody from the EBT told me.” They instead learned about this through word of mouth. The participants shared many ideas about how service providers and organizations can provide information to the community. This included having a centralized website to see when and where services and resources will be, a link to set up an appointment, and a function to search by location for resources specific to that area. It was also suggested that information about services, resources, and events should be posted on bulletin boards in community areas and parks and that door-knocking with information about resources and events could help. One participant stated that organizations should “have a presence in the neighborhood…cause there’s people that do not get out much or do not know these things” (Participant 21). Other information-sharing ideas included engaging in preventative information sharing at schools, handing out fliers at church, and encouraging information sharing by word of mouth.

#### 3.3.2 Technology

Participants noted difficulties with using or having regular access to technology needed to obtain services (e.g., booking appointments online). Participant 14 shared that “Everything is done through technology and not all of us have access to this technology.” The use of technology was noted as a particular barrier for older adults accessing information about services. Participant 20 shared their concern about organizations not reaching older adults, explaining: “We have to think, also, seniors do not know how to use the computer. So we have to help our seniors, and we are not doing that.” This participant suggested that posting signs in low-income senior housing buildings and sharing information in person instead of only online could help with this.

#### 3.3.3 Scheduling process and limited appointments

Multiple participants discussed challenges with scheduling processes, including long wait times and slow response times for getting an appointment. They also noted challenges with organizations offering only a limited number of appointments, particularly free clinics that fill up quickly. Participant 26 explained: “It’s like the second that word gets out, it’s full. Which I mean just speaks to the need, right?”

#### 3.3.4 Geographic location and transportation

Geographic location/proximity was also noted as a barrier. Participants shared that they often must travel long distances for services and that there are not enough service locations. They highlighted several challenges related to transportation, including the cost of transportation, relying on family members for transportation, and being unable to take their pets on public transportation. Participant 18 explained “…for those who live in some areas that do not have that help, or who do not have transportation. Sometimes people have to take the bus…Even then, trying to get to a bus… That can be hard, or they’ll miss it.” Participant 3 noted that not having a car has impacted their ability to access pet care services, such as taking their dogs to the groomers.

#### 3.3.5 Accommodations

Participants also noted a lack of accessibility for persons with a disability and/or persons requiring additional accommodations. They shared that there are challenges for people with mobility restrictions. A lack of service provider knowledge of rules and regulations related to service animals and emotional service animals was also noted, resulting in challenges with accessing transportation and housing.

#### 3.3.6 Documentation requirements

Several participants described documentation requirements for services that posed barriers to accessing resources, including proof of income, government identification, and other forms of documentation that are required to obtain housing, pet food, and some of the free/low-cost mental health supports.

#### 3.3.7 Lack of mental health support

A lack of availability and affordability of mental health support was noted by multiple participants. Participant 26 explained: “I have mental health struggles, and I make too much money to qualify for help through the State. But I do not make enough to pay for it myself.” Participant 4 shared that while they are enrolled in a mental health program, having only a half an hour visit with a doctor is not sufficient and that having 2 h with a doctor would be ideal (“Yo estoy en un programa de salud mental. Yo pienso que media hora un doctor contigo no es suficiente. Yo pienso que serían 2 horas estar contigo”).

### 3.4 Need for trusted providers

Participants were asked to share what individuals or organizations they trust to provide services for people or pets. Participants noted that a lack of trust in service providers is a barrier to accessing both human and pet well-being services, and shared that broken promises largely contribute to this lack of trust. They shared several interactions with organizations who promise services or resources, telling people, “We will help you with this,” and then never come back.

Participant 16 shared: “People in my community will not go [to clinics] because there’s been rescues keeping their animals… They’ll say do not worry we’ll get them spayed. They never came back.” This participant further explained how community members were hesitant or unwilling to work with one specific nonprofit animal welfare organization because of their lack of trust in other animal rescue organizations: “It took us time to build trust in that community. Just for these people to come and wipe it out in one fell swoop. In 1 day, stealing people’s animals. So that’s a broken promise.”

Participant 23 shared about how they navigated a lack of trust for the shelter in their community: “[W]e were homeless at the time. And we got a dog that it got to a point where we could not take care of the dog…and it broke my heart because the dog loved us so much. But we had to take it in. And you know, I could not just tell the truth, because…they were gonna charge me something that I did not have. Is it your dog? No, it’s a stray dog…. Maybe I did not have to lie. But you know, I wanted to make sure that she got in there.” The participant also shared that they feel “haunted” by this experience.

It was also noted that the disconnect and separation between organizational leadership and the people on the ground fuels distrust because the paperwork causes long waits to get resources to a person. Participant 15 explained: “The people on the ground see what they need. Look, this person needs socks. I’m gonna come back tomorrow with socks. But no, they have to fill out paper. It’s gonna take 2 weeks. And then by the time they really talk to that person, that person’s gone. And then that person thinks that that guy’s a liar, that’s it.”

Another participant shared about their lack of trust in elected officials. Participant 14 shared: “You get so disillusioned that you no longer want to invest your time in it. Why does not my voice matter? They tell you they are there for you, that they are your employees, you voted for them. But when it comes to choosing between what the community wants and the money, they are going to leave the city. They leave because of the money…And it is very frustrating that you want to make those changes, but every time you try…nothing happens.” (“[E]ste te desilusionas tanto que ya no quieres invertirle tu tiempo. Para qué mi voz no importa, le dicen que están ahí para ti, que son tus empleados, tú votaste por ellos. Pero cuando se trata de elegir entre lo que la comunidad quiere y el dinero, que va a dejar a la ciudad. Ellos se van por el dinero…Y es bien frustrante que tú quisieras hacer esos cambios, pero cada vez…que lo intentas nada pasa.”).

The participants also expressed their desire for organizations to report on the impact of their work, and for more information from organizations about how they are using their funding. They noted that follow-up after community-involved activities like focus groups is needed, and transparency regarding whether funds are going towards the needs of the community. Participant 14 noted that more monitoring would be helpful to ensure that funds are “really reflected in the community.”

The participants shared that due to accessibility issues and lack of trust in existing services, they often rely on family members and Google to find information about services and resources.

Participants named the following individuals or organizations in Los Angeles County as trusted providers of human and pet well-being information and services (listed in alphabetical order): Clancy’s Closet, Downtown Dog Rescue, Heart LA, “the local Humane Society,” Paws for Life, Penny Lane, Senator Alex Vadilla, Stay Housed LA, Stray Cat Alliance, and Terryl Daluz/Loving Paws. Another service that was noted was food banks and meal programs put on by local schools and organizations.

#### 3.4.1 Judgment and discrimination

Participants shared about their experiences of judgment and discrimination from both service providers and neighbors. This included experiences of the following: assumptions that those with illnesses cannot have or care for a pet; racism and discrimination regarding pet ownership; feeling targeted or stigmatized for their mental health and need for an emotional support animal; fear of talking about mental health with human service providers; negative assumptions from neighbors or landlords about cleanliness of pets or pet owners; invasive questioning by service providers about how they care for their pets; dog breed or size discrimination and assumptions from neighbors and landlords based on their dog’s breed and size (e.g., large dogs are uncontrollable and loud [Participant 1]; Pitbulls are dangerous [Participants 18 and 21]); and pet adoption discrimination, in particular judgments from shelters and rescue organizations about how they will care for their pets based on their race or ethnicity.

Participant 16 explained their experience of racial discrimination in the pet adoption process, sharing that they feel the need to prove themself as a pet owner to avoid being racially stereotyped. Speaking about the experience of being Hispanic and trying to adopt a dog, the participant shared: “. Especially if you are Hispanic. If you are Hispanic they look at you to see if you are actually taking care of your dog, because.there’s rescues who will look at you like ‘we are not gonna adopt to you.’” This participant also shared about the additional “hoops” they have to jump through to be seen as a responsible pet owner: “We have to go to all these extras to make sure that you are a viable owner, because they feel like Hispanics keep their dogs outside, and only outside. And that’s not true.”

#### 3.4.2 Trauma-informed services & safe spaces

Participants were asked to share about how service providers can be more mindful about the trauma that people in the community have experienced, and what makes them feel ‘safe’ when visiting a service provider. Participants shared that there is a lack of trauma-informed services, a lack of compassion and understanding, and a lack of continued support. Participants shared that they often feel treated like “just another case” (Participant 1), and noted their desire to feel heard and confident that they will not be turned away from services.

In general, participants shared the desire to be able to ask open and honest questions without feeling like they will be judged, embarrassed, or have negative repercussions because of asking the ‘wrong question’ or saying the ‘wrong thing’. Participant 26 shared about the particular need to feel safe talking to service providers about mental health: “I’ve never felt safe like going in and talking about mental health. I always felt hesitant to be fully honest with a lot of them, which is not helping me at all.”

Participant 16 noted that the lack of lived experience from service providers impacts how service providers understand and empathize with clients. This participant explained that most of the workers at a family housing program did not share their experience of being houseless, and that the workers who were formerly houseless were “the ones who got stuff done” and helped them learn how to navigate the systems.

Participants also shared many ideas of what characteristics and approaches service providers should embody to provide a safer space. This included:

- Understanding, listening, and having good communication skills;
- Having compassion and empathy;
- Having consideration and respect;
- Having cultural competence;
- Having patience;
- Meeting people where they are at in life;
- Practicing non-judgment;
- Being non-discriminatory;
- Being non-ableist;
- Providing real solutions;
- Hiring workers with lived experience;
- Truly caring about people and animals;
- Approaching situations with nuance;
- Avoiding the use of a one-size-fits-all approach;
- Offering a spectrum of care and treatment options;
- Offering alternatives to upfront payment

### 3.5 Housing

Participants shared about many challenges related to finding and keeping housing, including a lack of pet-friendly housing options; pet and child discrimination; pet breed, size, and weight restrictions; expensive pet deposits and fees; fear of losing housing or becoming homeless again; lack of access to information about housing and tenant rights; tensions with neighbors about pets; the power of landlords; proof of income and financial requirements; negative assumptions about pets; and a lack of services in SROs (single-room occupancy housing).

Many participants expressed that the intersection of a lack of access to information about housing and tenant rights and the power that landlords have creates a dangerous power dynamic: “I think that the lack of information gives [landlords] a lot more power…I feel like a lot of people do not even know their rights… Landlords bet on people renting to not know their rights,” (Participant 21). Participants also shared that it seems as though the rules and fees related to renting to pet owners are up to the landlord or owner’s discretion. To describe this, Participant 16 shared the example of landlords increasing the cost of a security deposit at their discretion when they do not want to rent to a person with a pet.

Regarding what housing-related information would be helpful to have access to, participants shared the following: general housing and tenant rights related to pets (“A breakdown of what your tenant rights are as a pet owner. And then, resources that are readily available for you.” [Participant 27]); what pet-related housing fees are legal; how many pets you can legally have in a home; limitations of landlord’s rights; legal support for rent and housing disputes; and more advertisements about resources.

### 3.6 Parks, green spaces, and environmental justice

Participants shared that they do not feel that they have adequate access to green spaces and natural areas (e.g., parks) in their communities. Participants also acknowledged that there are many nice parks and green spaces with amenities in wealthier neighborhoods. Of the parks and green spaces that do exist in their communities, participants noted safety concerns that prevent them from accessing these spaces. Participants expressed their shared desire to have more parks and green spaces within proximity (walking distance) that are both cat and dog-friendly (including off-leash areas), and accessible to people with mobility disabilities. On the topic of dog and cat-friendly parks and green spaces, Participant 16 shared “I need a dog park where I can take my cat or [a] cat park or something like that. There’s no dog parks in Compton, not one.”

Participants noted that the lack of access to green spaces has negative impacts on themselves, their families, and their pets. For example, Participant 27 shared: “It would be nice to take [my dog] to the park more, and I do feel like he’s always happier when he gets to go do those things.” Participant 18 highlighted the perceived benefits of parks and green spaces for pets and humans when they noted that “plants, nature or just greenery itself is what makes a certain place feel more alive. Not only just for the person, but also for your pet as well.” Participants also shared that they feel green spaces make a neighborhood cleaner, kinder, and happier (Participant 25) and that more grass and green spaces are good for the environment (Participant 27). Participant 1 shared: “I would take my dog to the park every day if I had a place that was closer, or within walking distance of my house.”

Participants also shared their thoughts about the connection between pet ownership and environmental justice issues in their communities, and how the environment impacts the health and well-being of their family and pets. Pet waste was a topic that many participants agreed was an issue in their communities. They shared that owners often do not pick up their pet’s waste, which impacts the environmental quality of their neighborhoods. To remedy this, they suggested that more waste bins are needed in parks and on sidewalks, for both general waste and pet waste. They also shared their desire to have a designated area for pets to use the bathroom. Participants shared that the pet waste bins and general waste bins that do currently exist are not regularly cleaned out. They also noted that the free dog waste bag stands that currently exist are often empty.

Participant 16 noted that the lack of portable bathrooms, hand wash stations, and waste bins in houseless communities causes both human and environmental health concerns. Participants also shared that they wish to receive more support from environmental health-related organizations to address environmental issues in their communities and their health-related impacts. For example, Participant 11 shared about the influx of companies negatively affecting their community with warehouses and toxic fumes.

### 3.7 Community care and mutual aid

Participants shared stories about how they engage in community care and mutual aid (mutual aid refers to community members supporting each other by sharing resources and/or services as a means of solidarity and to overcome barriers) due to the lack of accessibility of existing services. In particular, this included supporting community members/friends and taking the initiative to improve one’s community.

Participants shared that they will offer mutual aid and help others in their community with finding resources even if they do not have money to give them. Participant 3 shared that they make extra income to support their pets and community pets by collecting recyclables. Participant 16 explained that their lived experience and lack of support made them want to become a liaison and help others in similar situations. This participant volunteers their time to help with pets in the community because there are no other services or resources. Many of the participants shared they learn about resources from other people they know who have pets, community organizations, and flyers that are at churches. Regarding improving one’s own community, Participant 16 shared that their creative solution to address the lack of green spaces in their community is to plant their own greenery, but noted that they then “get dinged for it. You have too many trees in your yard.”

The theme of community care showed up in the focus groups themselves. In one focus group, after hearing about the barriers to accessing services that one participant was experiencing, two other participants offered rides and other solutions to support accessing transportation and offered to support the cost of veterinary care in creative ways. In the other focus groups, participants shared resources and information with each other.

## 4 Discussion

The focus group participants covered a number of topics when discussing how to improve health services and advance health equity for people and pets in Los Angeles County, CA. In the sections to follow, we will discuss the nine most prevalent concepts and their relevance for health services professionals: access to care; the human-animal bond; racism and discrimination; trauma-informed care; cultural responsiveness; mutual aid; dog parks, green spaces, and environmental justice; and housing and tenants rights.

### 4.1 Access to care

The topic of unequal access to veterinary care and pet support have been addressed extensively in several recent research studies, exploring what barriers to accessing care exist, how this lack of access impacts pet well-being, and what solutions can address these access barriers (11, 15). This study advances the research on access to care best practices for both the human and pet health sectors by providing detail about the barriers to accessing both human and pet care, and sharing stories about the harmful impacts of this lack of access, specifically in Los Angeles County, CA. The barriers that the participants in this study shared align with many of the barriers listed in other studies on access to care in both human and pet services, including affordability/financial limitations, geographical barriers, transportation, appointment availability, client identity, and preferred language.

This study highlights a desire and need for improved access to information. These findings reject a deficit view which typically suggest that inequities are from individual ‘deficiencies’. Rather, participants shared that they felt uninformed by service providers about how to access information on what services are available and where or when to access them. The participants also shared many practical suggestions for how service providers and organizations could provide information to the community in a more accessible way.

One tragic consequence of the lack of access to information and resources that was shared by participants is the separation of families, when pet owners have to make the impossible choice to surrender or re-home their pet. Improved access to information could help address this, but other issues such as a lack of pet-inclusive housing and shelters and the affordability of veterinary care and pet supplies must also be addressed. Support for programs such as temporary fostering and boarding can also help mitigate the possibility of family separation.

The experiences of participants in the current study also illustrated how the ability to access care for people and pets are interconnected. This interconnectedness can be explained through the social determinants of health, which are “the non-medical factors that influence health outcomes. They are the conditions in which people are born, grow, work, live, and age, and the wider set of forces and systems shaping the conditions of daily life” (32). However, the role of the social determinants of health and their impact on companion animal welfare have not been fully explored (33). There is an opportunity for future research and social service organizations to address the social determinants of health for people and pets through social care models, in order to improve access to care and achieve more equitable health outcomes (National (34)).

### 4.2 Human-animal bond

This study adds to the research exploring the bond between pets and BIPOC individuals by providing data that corroborates other research on the positive impact of the human-animal bond on human and pet well-being (35–37). The pet owners in the present study described pets as members of their family and source of mental, emotional, social, and physical health. Further, many participants in the present study expressed the belief that pet ownership is a fundamental right regardless of financial or housing status. Given the increasing prevalence of pet ownership in the U.S. and the impacts of the human-animal bond, health service professionals should consider collecting basic information regarding pets in the household. They may also play an integral role in assessing for risks and vulnerabilities related to basic needs for the pets.

To support health service professionals with collecting information regarding pets, there is a critical need for the development of equity-centered and culturally responsive assessment tools of pet ownership and the human-animal bond. Several scholars have highlighted the critical need for measures of the human-animal bond that include consideration of key health equity issues, including racial equity, economic and housing security, gender and sexual diversity, disability rights, and ecological justice (7, 18). For example, a study done by The Human Animal Bond Research Institute and Petco Love (38) represents one of the few examples of human-animal bond research in the U.S. that intentionally included BIPOC individuals in their dataset. The HABRI and Petco Love study (38), presented at the Association for Animal Welfare Advancement Conference in June 2022, found that the human-animal bond is strong across all ethnicities, and that pets have positive impacts on the community. However, several of the measures included in this human-animal bond instrument (e.g., “Nothing would ever convince me to give up my pet” and “If my pet needed extensive veterinary care, I would pay for whatever it takes”) fail to recognize the influence of systemic discrimination and other structural conditions, such as social determinants of health, on pet care giving practices. The pet-related assessment tools for health service professionals’ use should be developed in alignment with the call to shift evaluation measures from an “individualistic lens” that stigmatizes individuals for being vulnerable to systemic discrimination to an “equity lens” that recognizes the structural conditions and power dynamics that create vulnerability (39). Instead, other measures of the human-animal bond that are unbiased and value-neutral should be used, and these measures should not be predicated on an individual’s financial or socioeconomic status.

It is also important to acknowledge here that alongside the positive impacts, there are tradeoffs associated with pet ownership. Stressors related to pet ownership occur both on an individual level (40) and on a systemic level (41). For example, research conducted by Matijczak et al. (40). investigated the benefits and risks associated with living with companion animals for LGBTQ+ youth in the United States. Further, a recent study conducted by PetSmart Charities and Gallup (41) explored the costs of veterinary care and found that across income levels, financial barriers were cited as a reason to forgo or decline veterinary care–71% of pet parents who skipped or declined care report financial considerations as the reason.

Herzog (42) reports that while some studies have found that pet owners are better off regarding emotional and physical well-being when compared to non-pet owners, other studies have concluded that this is not the case. Herzog (42) notes that the reasons for the discrepancies between what many pet owners believe about the positive impact of pets and what the research findings demonstrate are unclear. Future research can better explore the positive and negative impacts of pet ownership by utilizing consistent and replicable methods amongst broader populations, and developing a pet ownership survey tool that can be used in these contexts, as this could allow for a better understanding of the relationship between pet ownership and mental health (43).

### 4.3 Racism and discrimination

Racism and discrimination continue to be critical drivers of racial health inequities in the United States. The focus group participants shared how their own experiences of racism and discrimination impacted their ability to access both human and pet care and in turn, affected both human and pet well-being. This study can advance the conversation on dismantling the racism and discrimination in both human and pet well-being services by highlighting specific lived experiences and solutions proposed by Los Angeles County pet owners that demonstrate that this is more than an individual provider issue.

The research literature has extensive documentation of the ways in which racism in human health services significantly contributes to health inequities by creating and perpetuating disparities in access to healthcare, quality of treatment, and overall health outcomes. These disparities persist across multiple levels of influence—individual, interpersonal, community, and societal (44). Scholars and practitioners note that achieving health equity is not possible without addressing the disparities that exist (45). As such, we must not only work to address and remove the barriers to access that are rooted in inequities, but also make efforts to understand the systemic roots of these disparities. Most research to date on racial equity in the animal welfare and protection industry has focused on implicit and explicit bias. For example, a study completed by Companions and Animals for Reform and Equity (8) found that animal welfare practitioners have a “moderate-to-strong implicit preferences for White people over Black people, Non-Hispanic people over Hispanic people, and rich people over poor people.” However, addressing implicit and explicit bias alone will not be sufficient for dismantling the health and well-being disparities for BIPOC families and their pets.

While bias intervention at the individual and provider level is an important step, we cannot just focus on addressing ‘unconscious’ and ‘implicit’ biases, as this approach removes personal responsibility and accountability for perpetuating harm and veils the reality that racism was and continues to be embedded in the foundation of U.S. society. In their exploration of the role of explicit and implicit biases in health care, Vela et al. (46) suggest that for provider-level bias interventions to succeed in improving health outcomes, structural inequities both inside and outside the system at hand must be addressed. As Boyd et al. (47) explains: “Obfuscating the role of racism in driving health inequities also gives frames such as implicit bias undue traction. This stalls progress to end inequities by entreating clinicians to tame ‘unconscious beliefs,’ rather than confronting explicit practices that undergird systemic inequities.” As such, interventions are needed that address biases at both the interpersonal and institutional levels. In particular, racial diversity in the animal welfare and protection sector workforce must be addressed. Further, there is an opportunity and need to develop a conceptual map of structural racism in the pet well-being service systems, similar to the map developed by Furtado et al. (48), demonstrating the impact of structural racism in pet services on both human and pet well-being.

### 4.4 Trauma-informed care

The findings from this study demonstrate community members’ desire for more trauma-informed and culturally responsive services for both people and pets. Trauma-informed care (TIC) is defined as “an approach to engaging people with histories of trauma that recognizes the presence of trauma symptoms and acknowledges the role trauma has played in their lives,” (49). The practice of implementing TIC is centered on “delivering services to clients in a way that is appropriate and sensitive to the unique needs” of those who have experienced trauma (50). Trauma-informed approaches have been utilized in a variety of fields, including child welfare and protection, nursing, domestic violence services, mental health services, and substance use services. In recent years, discussion about the use and benefits of trauma-informed approaches for the animal welfare and protection field has emerged (49–51). Incorporating trauma-informed approaches into animal welfare and protection services presents an opportunity to provide more equitable services that can improve outcomes for both people and pets. The findings from this study demonstrate that, from a community member perspective, utilizing trauma-informed approaches in animal welfare and protection service delivery could foster a safer space, increase trust, and improve accessibility of services.

Calderon de la Barca et al. (52) importantly note that trauma impacts people both individually and collectively. They explain that “[h]umanity is submerged in layers of individual, intergenerational, and collective trauma, but we generally do not recognize it. This prevents us from addressing the roots of collective challenges we face and keeps us from taking steps toward healing that can transform the systems around us,” (52). As such, both human and pet well-being service providers must find ways to address and uplift both individual and collective healing. Collective responses to trauma might include human and pet health service professionals spending time with and in communities to build relationships, creating community feedback and input mechanisms to foster trust and transparency, developing community-based programs that are rooted in trauma-informed approaches, and implementing processes that ensure follow-through and promise keeping.

When asked to share about how service providers can be more mindful of the trauma that people in the community have experienced and what makes them feel ‘safe’ when visiting a human or pet service provider, participants listed attributes and approaches (see Section 3.4.2) that directly align with the existing core trauma-informed principles. These core principles are acknowledgement and recognition that trauma is pervasive; safety; trust; choice and control; compassion; collaboration; and a strengths-based approach (53).

### 4.5 Cultural responsiveness

Cultural responsiveness is also considered to be a key principle of a trauma-informed approach (51, 54) and has been used to assess the effectiveness of a community-based animal welfare program (55). Participants in this study indicated multiple barriers to accessing care that are rooted in bias, discrimination and a lack of cultural competence on behalf of the provider. The participants shared that being culturally aware, competent, and non-discriminatory was important to them when reflecting on what makes a service provider ‘safe’. For example, Participant 18 explained: “When I will see a service provider, not just for myself, but like for my pets, I feel like somebody who’s gonna give me input on…Let us say, my dog needs some sort of medical treatment or something. Somebody who’s gonna give me input on what I should do and what I should provide for my pet, but also them taking into consideration what I think. Because, let us say I have…something about my beliefs or traditions that I should not take a certain thing, and they take that into account and just be mindful of what I want, not only for myself, but for my pet. I think that’s just what a safe provider would be for me.”

When these attributes of cultural responsiveness were not present within an organization’s service delivery model, community members shared that they felt hesitancy in engaging with them and trusting them with their pets. Several scholars have discussed the responsibility service providers have for addressing the mistrust in the US healthcare system (47). In particular, they underscore that it is vital to acknowledge that this mistrust is not the cause of inequitable access to care, rather, the inequitable access to care drives the mistrust. As Boyd et al. (47) explain, “While patient trust certainly shapes health care use behaviors and is an important part of the patient-physician relationship, incessant racial health inequities across nearly every major health index reveal less about what patients have failed to feel and more what systems have failed to do.”

In the context of animal welfare and protection, the positive impacts of addressing structural racism by addressing common barriers for accessing pet services have been documented. For example, Decker Sparks et al. (14) found that “when veterinary and animal welfare organizations deliberately remove structural barriers embedded with racial inequities, individuals, regardless of race and ethnicity, proceed with companion-animal sterilization.”

### 4.6 Mutual aid

In the presence of harmful, inaccessible, and inequitable systems, resilience is often found through community-led solutions, such as mutual aid and community care. Khan, Iwai, and DasGupta (19) explain that “throughout history, communities have found ways to heal and care for one another outside of institutional structures through mobilizing resistance, mutual aid, and collective care networks,” (p. 242). They go on to suggest that institutional structures such as health services can learn from these examples in order to expand their vision of “what counts as good health care” and that “the key is for health care and medical education to be willing to recognize these movements as central—not peripheral—to any broader vision of health justice,” (p. 242). The research participants spoke to the ways in which this concept of mutual aid has shown up in their own lived experiences and may be applied in broader human and pet well-being systems. The participants shared about the presence and importance of mutual aid and collective care in marginalized communities and highlighted many examples of how they support each other and engage in community care when faced with a lack of accessibility of services.

In a related discussion on community care, scholars have discussed how looking to the wisdom and lived experiences of neighborhood leaders and community activists and “working with these experts to address upstream realities and to collectivize structurally competent care” (p. 243) presents a path to a more racially just future (19). In other disciplines examples of investing in collective care include public health partnerships with community health workers, proximate leaders, and promotoras (promotores, or promotoras de salud), is a Spanish term to describe trusted individuals who empower their peers through education and connections to health and social resources in Spanish speaking communities (56). Participants expressed their support for these models for care. For example, one participant expressed that they had more positive and helpful social service experiences when working with staff who had lived experiences similar to theirs.

### 4.7 Dog parks, green spaces, and environmental justice

The built environment within any community is an influential social determinant of health. Several scholars have explored the disparities in access to green spaces in the U.S., observing that there is an inequitable distribution of green spaces by socioeconomic status and race/ethnicity (57–59). Kephart (57) explains that “studies consistently demonstrate an association between racial residential segregation and less exposure to tree canopy coverage, vegetation, and parks.” In addition, when BIPOC individuals do have “greater access to parks, these parks tend to be more congested and contain less amenities than parks located in areas with predominantly [w]hite residents” (57). The findings from this study echo these conclusions. Participants indicated that they observed a stark difference in the prevalence of green spaces and dog parks in their lower-income communities, compared to higher-income communities in their city. They also noted that the green spaces and dog parks that do exist in their communities have little to no amenities (e.g., water fountains, benches, garbage bins) and that cleanliness and safety were issues that often prevent them from utilizing these spaces. Many participants also shared that they felt their pet’s well-being would increase if they had better access to green spaces and dog parks.

### 4.8 Housing and tenants rights

Previous literature documents a host of housing-related challenges for families that rent with pets, including: size, weight, and breed restrictions; the constant threat of housing insecurity; expensive pet deposits and fees, which impact the accessibility of safe and affordable housing; and the long-history of landlord-friendly laws that empower landlords to discriminate and mistreat tenants and potential tenants, especially those with pets (12, 60–62). Focus group participants shared about how many of these same challenges related to finding and keeping housing that is inclusive of their pet are prevalent in Los Angeles County. Participants discussed the impacts of pet, breed, size, and weight restrictions, expensive pet deposits and fees, fear of losing housing or becoming houseless again, lack of access to information about housing and tenant rights, and the unequal power dynamics between tenants and landlords.

The findings from this study align with previous research conducted by Rose et al. (61), which examined pet-friendly rental housing throughout Forsyth County, North Carolina, a predominantly Black area. The study found evidence of racial inequalities by neighborhood and housing discrimination in Winston-Salem, North Carolina based on pet policies in housing. In predominantly white neighborhoods it was much easier for people with companion animals to find housing even in predominantly white low-income neighborhoods (61). Applebaum et al. (12) built upon the work of Rose et al. (61) and found that within Texas, the costs associated with housing a family with a pet disproportionately harmed economically disadvantaged populations. Communities with a higher percentage of white residents were found to have a lower pet fee burden compared to communities with more people of color. The study also showed that the pet fee burden was particularly pronounced for Latino/a/e communities and that marginalized groups were more likely to experience this burden because of the discrimination they face beyond pet ownership and the lack of additional resources to dedicate to pet fees. Focus group participants shared their lived experiences with pet fees, and advocated for a change in pet-related policies and regulations, as well as access to legal aid and financial support services.

The focus group participants discussed how restrictive pet policies like breed restrictions, weight restrictions and no-pet policies negatively impact Los Angeles County residents and shared their fears and concerns related to potentially losing their existing housing or becoming homeless again. These findings align with previous research that measured how the threat of eviction and homelessness negatively impacts a person’s physical and mental health (63, 64). Benfer et al. (63) indicated that people facing eviction are more likely to suffer from higher mortality, respiratory conditions, high blood pressure, poor self-rated general health, coronary heart disease, sexually transmitted infections, and drug use. Eviction also impacts their mental health, often leaving marginalized individuals with depression, anxiety, mental health hospitalization, exposure to violence, and suicide without the resources to treat their issues. These findings highlight the critical need for reform in pet-related housing policies in order to safeguard housing security and support the overall health and well-being of marginalized people and their pets.

There are several organizations focused on this issue of advancing pet-inclusive housing for renters. For example, Housing Equity and Advocacy Resource Team (HEART) Los Angeles, is a nonprofit public interest law firm dedicated to addressing the critical challenge in the Los Angeles area of helping low-income individuals, families, and their pets maintain stable housing. Michelson Found Animals’ Pet Inclusive Housing Initiative (PIHI) is a program focused on removing barriers that prevent renters from keeping pets in their homes–PIHI supports this change by providing meaningful resources to housing stakeholders to help keeps pets and their families together in housing, and they have conducted valuable research underscoring the benefits of increasing the number of pet-inclusive housing units to property owners and other housing providers. Supporting these housing equity-focused organizations is crucial in working towards pet-inclusive housing for all.

### 4.9 Limitations

This study had some limitations that can be addressed in future research. First, the findings from this research are community-specific and therefore, the generalizability of the findings to the broader population cannot be guaranteed. Second, our sample population included 81% of Hispanic/Latino/a individuals, 15% of Black/African American individuals, 4% of Native American/American Indian/Indigenous individuals, and 4% of White, non-Hispanic individuals. This is in comparison to the general population of Los Angeles County which is comprised of 49% Hispanic/Latino/a individuals, 9% Black/African American individuals, 1.5% Native American/American Indian/Indigenous individuals, 16% Asian individuals, and 25% White/non-Hispanic individuals (65). While we have overrepresentation of several BIPOC race/ethnicities in our sample, representation from Asian individuals is missing from this study’s sample population.

Further, using focus groups as a data collection method creates the possibility for certain biases to occur. Common biases related to focus groups include “dominance effect (a dominant individual shapes the discussion), halo effect (the perceived status of a group member influences the discussion), [and] groupthink (the members in a group tend to think similarly to maintain group cohesion)” (66). To minimize the impacts of these potential biases, the research team took steps to create a comfortable and safe environment where participants were encouraged to share their honest opinions. They did this by outlining the group expectations at the outset of the focus groups (e.g., respect each other’s opinions even if they differ), and the facilitators shared about themselves in an effort to build rapport and trust.

### 4.10 Conclusion

This study identifies community-specific priorities for improving health for people and pets in Los Angeles County, CA, as determined by community members themselves. The next step is to work with community partners in human and pet health services to co-create community-specific policies and programs based on this data that will advance health equity for people and pets in Los Angeles County, CA. The findings from this study both support and add to the current research on many topics within the scope of human and pet well-being and equitable access to care.

Further, the methodological approach of CPBR utilized in this study demonstrates the importance of prioritizing the perspectives of those with lived experience in the development and evaluation of programs and policies. Future research and evaluation projects should embody a commitment to mutually beneficial relationships, transparency, and “emancipatory methods that include community partnership” as opposed to the typical approach where researchers ‘parachute’ in and out (67). Researchers can draw on methodologies such as “culturally responsive evaluation, research, and pedagogy; feminist, Indigenous, and critical methodologies; community-based participatory research; and theories of social transformation, liberation, and racial justice” (68) in order to take on a more equity-centered research approach.

## Data Availability

The raw data supporting the conclusions of this article will be made available by the authors, without undue reservation.

## References

[1] American Pet Products Association [APPA]. (2024). Strategic insights for the pet industry, pet owners 2023 & beyond. Available online at: https://www.americanpetproducts.org/research-insights/appa-national-pet-owners-survey [Accessed October 1, 2024].

[2] Pew Research Center. (2023). About half of U.S. pet owners say their pets are as much a part of their family as a human member. Available online at: https://www.pewresearch.org/short-reads/2023/07/07/about-half-us-of-pet-owners-say-their-pets-are-as-much-a-part-of-their-family-as-a-human-member/ [Accessed October 1, 2024].

[3] Barr HK Guggenbickler AM Hoch JS Dewa CS. Examining evidence for a relationship between human-animal interactions and common mental disorders during the COVID-19 pandemic: a systematic literature review. Front Health Serv. (2024) 4:1321293. doi: 10.3389/frhs.2024.1321293, PMID: 38385049 PMC10879592

[4] Beetz A Uvnäs-Moberg K Julius H Kotrschal K. Psychosocial and psychophysiological effects of human-animal interactions: the possible role of oxytocin. Front Psychol. (2012) 3:234. doi: 10.3389/fpsyg.2012.00234, PMID: 22866043 PMC3408111

[5] Brooks HL Rushton K Lovell K Bee P Walker L Grant L. The power of support from companion animals for people living with mental health problems: a systematic review and narrative synthesis of the evidence. BMC Psychiatry. (2018) 18:31. doi: 10.1186/s12888-018-1613-2, PMID: 29402247 PMC5800290

[6] Gordon EW. Beginning with the end in mind: creating a practice that centers equity - part 2. Vet Clin N Am Small Anim Pract. (2024) 54:959–75. doi: 10.1016/j.cvsm.2024.07.018, PMID: 39266442

[7] Hawes SM Hupe T Morris KN. Punishment to support: the need to align animal control enforcement with the human social justice movement. Animals. (2020) 10:1902. doi: 10.3390/ani10101902, PMID: 33081392 PMC7602950

[8] Companions and Animals for Reform and Equity [CARE] (2021). Harvard project implicit results: implicit bias in animal welfare. Available online at: https://careawo.org/wp-content/uploads/2021/07/ProjectImplicited.pdf [Accessed October 1, 2024].

[9] Brown SE. The under-representation of African Americans in animal welfare fields in the United States. Anthrozoös. (2015) 18:98–121. doi: 10.2752/089279305785594225

[10] Campbell A. Matz K. Hawes S. (2024). Revealing animal well-being’s economic glass ceiling: the impact, challenges, and opportunities of BIPOC pet businesses. Research Gurus & Companions and Animals for Reform and Equity (CARE). Available online at: https://careawo.org/rd/ [Accessed November 21, 2024].

[11] Pasteur K Diana A Yatcilla JK Barnard S Croney CC. Access to veterinary care: evaluating working definitions, barriers, and implications for animal welfare. Front Vet Sci. (2024) 11:1335410. doi: 10.3389/fvets.2024.1335410, PMID: 38304544 PMC10830634

[12] Applebaum JW Horecka K Loney L Graham TM. Pet-friendly for whom? An analysis of pet fees in Texas rental housing. Front Vet Sci. (2021) 8:1279. doi: 10.3389/fvets.2021.767149, PMID: 34820439 PMC8606550

[13] Cardona A Hawes SM Moss L Merasty J Connolly K O’Reilly K. Perspectives on free-roaming dogs on the tribal lands of the three affiliated tribes at Fort Berthold, North Dakota. Animals. (2023) 13:1422. doi: 10.3390/ani13081422 37106984 PMC10135030

[14] Decker Sparks JL Camacho B Tedeschi P Morris KN. Race and ethnicity are not primary determinants in utilizing veterinary services in underserved communities in the United States. J Appl Anim Welf Sci. (2018) 21:120–9. doi: 10.1080/10888705.2017.1378578, PMID: 28960091

[15] Hawes SM Hupe TM Winczewski J Elting K Morris KN Arrington A. Measuring changes in perceptions of access to pet support care in underserved communities. Front Vet Sci. (2021) 8:745345. doi: 10.3389/fvets.2021.745345 34957275 PMC8702831

[16] LaVallee E Mueller MK McCobb E. A systematic review of the literature addressing veterinary care for underserved communities. J Appl Anim Welf Sci. (2017) 20:381–94. doi: 10.1080/10888705.2017.1337515, PMID: 28657796

[17] Mascitelli TM Graham TM Loney L Applebaum JW Murray CM Binns-Calvey M. Barriers to finding and maintaining pet-inclusive affordable housing: tenant experiences in Houston, Texas. Front Vet Sci. (2024) 11:1465682. doi: 10.3389/fvets.2024.1465682 39529854 PMC11551556

[18] Jenkins JL Rudd ML. Decolonizing animal welfare through a social justice framework. Front Vet Sci. (2022) 8. doi: 10.3389/fvets.2021.787555 PMC8829506 35155644

[19] Khan ZH Iwai Y DasGupta S. Abolitionist reimaginings of health. Am Med Assoc J Ethics. (2022) 24:E239–46. doi: 10.1001/amajethics.2022.239, PMID: 35325526

[20] Israel BA Schulz AJ Parker EA Becker AB. Review of community-based research: assessing partnership approaches to improve public health. Annu Rev Public Health. (1998) 19:173–202. doi: 10.1146/annurev.publhealth.19.1.173, PMID: 9611617

[21] Fleming PJ Stone LC Creary MS Greene-Moton E Israel BA Key KD. Antiracism and community-based participatory research: synergies, challenges, and opportunities. Am J Public Health. (2023) 113:70–8. doi: 10.2105/AJPH.2022.307114, PMID: 36516389 PMC9755941

[22] Israel BA Coombe CM Cheezum RR Schulz AJ McGranaghan RJ Lichtenstein R. Community-based participatory research: a capacity-building approach for policy advocacy aimed at eliminating health disparities. Am J Public Health. (2010) 100:2094–102. doi: 10.2105/AJPH.2009.170506, PMID: 20864728 PMC2951933

[23] Copeland V Wells R. ‘The real data set’: A case of challenging power dynamics and questioning the boundaries of research production. Mich J Commun Serv Learn. (2024) 30:3676. doi: 10.3998/mjcsl.3676

[24] Chicago Beyond. (2019). Why am I always being researched? Available online at: https://chicagobeyond.org/insights/philanthropy/why-am-i-always-being-researched/ [Accessed December 3, 2024]

[25] Esposito J Evans-Winters VE. Introduction to intersectional qualitative research SAGE Publications, Inc (2022).

[26] Tapp H De Hernandez BU Smith H. Community-based participatory focus groups In: Editors Matthes J Davis CS Potter RF. The international encyclopedia of communication research methods. Wiley (2017). 1–3.

[27] National Health Council (2024). Patient compensation tools. Available online at: https://nationalhealthcouncil.org/access-the-fmv-calculator (Accessed October 23, 2024).

[28] Creswell JW Poth CN. Qualitative inquiry and research design: Choosing among five approaches. Fourth ed SAGE Publications (2016).

[29] McKenna L. Translation of research interviews: do we have a problem with qualitative rigor? Nurse Author Ed. (2022) 32:1–3. doi: 10.1111/nae2.31

[30] Santos HPO Black AM Sandelowski M. Timing of translation in cross-language qualitative research. Qual Health Res. (2015) 25:134–44. doi: 10.1177/1049732314549603, PMID: 25189538

[31] Abfalter D Mueller-Seeger J Raich M. Translation decisions in qualitative research: a systematic framework. Int J Soc Res Methodol. (2020) 24:469–86. doi: 10.1080/1365579.2020.1805549

[32] World Health Organization. (2024). Social determinants of health. Available online at: https://www.who.int/health-topics/social-determinants-of-health#tab=tab_1 [Accessed October 23, 2024].

[33] McDowall S Hazel SJ Chittleborough C Hamilton-Bruce A Stuckey R Howell TJ. The impact of the social determinants of human health on companion animal welfare. Animals. (2023) 13:1113. doi: 10.3390/ani13061113, PMID: 36978653 PMC10044303

[34] National Academies of Sciences, Engineering, and Medicine. Integrating social care into the delivery of health care: Moving upstream to improve the nation's health. Washington, DC: The National Academies Press (2019). 31940159

[35] Risley-Curtiss C. Social work practitioners and the human—companion animal bond: a national study. Soc Work. (2010) 55:38–46. doi: 10.1093/sw/55.1.38, PMID: 20069939

[36] Risley-Curtiss C Holley LC Wolf S. The animal-human bond and ethnic diversity. Soc Work. (2006a) 51:257–68. doi: 10.1093/sw/51.3.257, PMID: 17076123

[37] Risley-Curtiss C Holley LC Cruickshank T Porcelli J Rhoads C Bacchus DNA. “She was family” women of color and animal-human connections. Affilia. (2006b) 21:433–47. doi: 10.1177/0886109906292314

[38] Human Animal Bond Research Institute and Petco Love. (2022). Understanding the role of race and ethnicity in pet ownership and care. Available online at: https://habri.org/DEI [Accessed October 23, 2024].

[39] American Medical Association and Association of American Medical Colleges’ Center for Health Justice [AMA & AAMC]. (2021). Advancing health equity: A guide to language, narratives and concepts. Available online at: https://www.ama-assn.org/system/files/ama-aamc-equity-guide.pdf [Accessed October 1, 2024].

[40] Matijczak A McDonald SE Tomlinson CA Murphy JL O’Connor K. The moderating effect of comfort from companion animals and social support on the relationship between microaggressions and mental health in LGBTQ+ emerging adults. Behav Sci. (2021) 11:1. doi: 10.3390/bs11010001, PMID: 33374678 PMC7822483

[41] PetSmart Charities Gallup (2025). State of pet care study: pet parents' assessment of American veterinary care Available online at: https://www.gallup.com/analytics/659123/gallup-petsmart-charities.aspx (Accessed May 16, 2025).

[42] Herzog H. (2021). Are pets as good for us as we think they are? Psychology Today. Available online at:https://www.psychologytoday.com/us/blog/animals-and-us/202109/arepets-good-us-we-think-they-are (Accessed May 16, 2025).

[43] Scoresby KJ Strand EB Ng Z Brown KC Stilz CR Strobel K. Pet ownership and quality of life: a systematic review of the literature. Vet Sci. (2021) 8:332. doi: 10.3390/vetsci8120332, PMID: 34941859 PMC8705563

[44] National Institute on Minority Health and Health Disparities. (2017). NIMHD research framework. Available online at: https://nimhd.nih.gov/researchFramework [Accessed October 23, 2024]/

[45] Webb Hooper M Pérez-Stable EJ. Health equity is not possible without addressing disparities. Health Psychol. (2023) 42:625–7. doi: 10.1037/hea0001306, PMID: 37589700

[46] Vela MB Erondu AI Smith NA Peek ME Woodruff JN Chin MH. Eliminating explicit and implicit biases in health care: evidence and research needs. Annu Rev Public Health. (2022) 43:477–501. doi: 10.1146/annurev-publhealth-052620-103528, PMID: 35020445 PMC9172268

[47] Boyd R.W. Lindo E.G. Weeks L.D. McLemore M.R. (2020). On racism: a new standard for publishing on racial health inequities. Available online at: https://www.healthaffairs.org/content/forefront/racism-new-standard-publishing-racial-health-inequities [Accessed October 1, 2024]

[48] Furtado K. Verdeflor A. Waidmann T.A. (2023). A conceptual map of structural racism in health care. Urban Institute. Available online at: https://www.urban.org/research/publication/conceptual-map-structural-racism-health-care [Accessed October 1, 2024]

[49] Dorst L Dodson D McKee B. A multidisciplinary and trauma-informed approach to community-based support programs. IAABC Found J. (2024) 29. doi: 10.55736/iaabcfj29.1

[50] Stevenson R Morales C. Trauma in animal protection and welfare work: the potential of trauma-informed practice. Animals. (2022) 12:852. doi: 10.3390/ani12070852, PMID: 35405841 PMC8997134

[51] Morales C. Stevenson R. (2021). Helping people and animals together: taking a trauma-informed, culturally safe approach towards assisting placed-at-risk people with addressing animal neglect. Vancouver Humane Society.

[52] Calderon de Barca L. Milligan K. Kania J. (2024). Healing systems. Stanford Social Innovation Review. Available online at: https://ssir.org/articles/entry/healing-trauma-systems [Accessed October 1, 2024].

[53] Manitoba Trauma Information and Education Center. The trauma-informed toolkit. 2nd ed Klinic Community Health Care Centre (2013).

[54] Substance Abuse and Mental Health Services Administration [SAMHSA]. (2014). SAMHSA’S concept of trauma and guidance for a trauma-informed approach. Available online at: https://store.samhsa.gov/product/samhsas-concept-trauma-and-guidance-trauma-informed-approach/sma14-4884 [Accessed October 11, 2024].

[55] Gandenberger J Hawes SM Wheatall E Pappas A Morris KN. Development and initial validation of the animal welfare cultural competence inventory (AWCCI) to assess cultural competence in animal welfare. J Appl Anim Welf Sci. (2023) 26:540–51. doi: 10.1080/10888705.2021.2008934, PMID: 34894907

[56] MHP Salud. (2024). Promotores and promotoras de salud. Available online at: https://mhpsalud.org/our-programs/promotoras-de-salud/ [Accessed October 22, 2024].

[57] Kephart L. How racial residential segregation structures access and exposure to greenness and green space: a review. Environ Just. (2022) 15:204–13. doi: 10.1089/env.2021.0039

[58] Klompmaker JO Hart JE Bailey CR Browning MH Casey JA Hanley JR. Racial, ethnic, and socioeconomic disparities in multiple measures of blue and green spaces in the United States. Environ Health Perspect. (2023) 131:017007. doi: 10.1289/EHP11164 36696102 PMC9875842

[59] Robinson T Robertson N Curtis F Darko N Jones CR. Examining psychosocial and economic barriers to green space access for racialised individuals and families: a narrative literature review of the evidence to date. Int J Environ Res Public Health. (2022) 20:745. doi: 10.3390/ijerph20010745, PMID: 36613069 PMC9819928

[60] Chen KL Miake-Lye IM Begashawm MM Zimmerman FJ Larkin J McGrath EL. Association of promoting housing affordability and stability with improved health outcomes: a systematic review. JAMA Netw Open. (2022) 5:1–15. doi: 10.1001/jamanetworkopen.2022.39860 PMC9631101 36322083

[61] Rose D McMillian Carter O. Pet-friendly rental housing: racial and spatial inequalities. Space Cult. (2020):1–14. doi: 10.1177/1206331220956539

[62] Uhlig A Bellamy W Amos M Bernstein D Brause J Morin E. Preventing eviction and housing loss: taking advantage of a one health approach and the human-companion animal bond. J Health Care Law Policy. (2023) 26:181–214.

[63] Benfer EA. Eviction, health inequity, and the spread of COVID-19: housing policy as a primary pandemic mitigation strategy. J Urban Health. (2021) 98:1–12. doi: 10.1007/s11524-020-00502-1, PMID: 33415697 PMC7790520

[64] Swope C Hernández D Yoon L (2023). The four pillars of housing influencing health equity. National Collaborating Centre for Environmental Health. Available online at: https://ncceh.ca/resources/blog/four-pillars-housing-influencing-health-equity [Accessed October 1, 2024].

[65] U.S. Census Bureau. (2024). Los Angeles County, California. Available online at: https://www.census.gov/quickfacts/fact/table/losangelescountycalifornia/RHI725223 [Accessed May 15, 2025].

[66] Nyumba O Wilson K Derrick CJ Mukherjee N. The use of focus group discussion methodology: insights from two decades of application in conservation. Methods Ecol. (2018) 9:20–32. doi: 10.1111/2041-210X.12860, PMID: 40421581

[67] Lett E Adekunle D McMurray P Asabor EN Irie W Simon MA. Health equity tourism: ravaging the justice landscape. J Med Syst. (2022) 46:17. doi: 10.1007/s10916-022-01803-5, PMID: 35150324 PMC8853313

[68] Venkateswaran N Feldman J Hawkins S Lewis MA Armstrong-Brown J Comfort M. Bringing an equity-centered framework to research: Transforming the researcher, research content, and practice of research. Research Triangle Park, NC: RTI Press (2023). 37289917

