# Supplementary material for: Centering community voices: advancing health equity for people and pets in Los Angeles County through community-based participatory research
Source: Front Vet Sci. 2025 Jun 18;12:1539811. doi: 10.3389/fvets.2025.1539811 (PMC12218251; doi:10.3389/fvets.2025.1539811)
Supplement: Supplementary file 1 [file Table_1.docx]

**Table 1. Focus Group Questions**

***Icebreaker***

- What do you love about living in Los Angeles?
- Do you have any pets? If yes, please use one word to describe your pet.

***Pets and Human Wellbeing***

1. Do you feel that your pet(s) have a positive impact on your health and overall well-being? On your mental health? On your perception of safety and security in your neighborhood? On your social connections?
   1. If yes, please share a story about a time where your pet had a positive impact on your health and overall well-being.
2. Have you ever felt judged or stigmatized for being a pet owner in your community?
3. Do you believe that pet ownership is a fundamental right for all individuals, regardless of income level?

***Vision for Human and Pet Wellbeing in Los Angeles***

1. What does a healthy and equitable community look like for your family and your pets (for example, access to services for people and pets, green spaces, affordable housing in areas with resources for kids and pets)?
   1. If you were to design a pet-inclusive living environment, what would it look like?
2. What are the most important human or animal well-being items or services that you want and/or need community services or nonprofits in your area to provide?

***Current Human and Pet Wellbeing Services***

1. What community services and nonprofit programming already exists in your community to support families and their pets? What is missing/what changes are needed?
2. Who do you trust for information about caring for your pet and your family? This could be individuals in your community and/or specific community services or nonprofit organizations.

***Experience with Service Providers***

1. Tell us a story of a time you experienced barriers to accessing services for yourself (healthcare, mental health, social services, food, transportation, etc.) or your pets (veterinary care, behavior training, grooming, food, etc.).
   1. For example, the cost of services is too high, services are not available in your preferred language, proof of income is required for services, etc.
2. Have you ever felt judged or discriminated against by your human or animal community services or nonprofit programming provider? Has that feeling of judgment impacted your decision-making in any way?
3. Can you share about a time when you felt that a community service/nonprofit program broke a promise?
4. How can community services/nonprofit programs be more mindful about the trauma that people in the community have experienced? What does an organization or person acting in a trauma-informed way mean to you?
5. What makes you feel ‘safe’ when visiting a service provider? What does a ‘safe space’ mean to you?

***Housing***

1. Have you ever experienced challenges with finding or keeping housing for you and your pets?
2. What information or services are/would have been most impactful for finding or keeping housing?

***Environmental Justice***

1. Do you think there is a connection between pet ownership and environmental justice issues in your community?
   1. For example, how do you think pet ownership affects the overall environmental quality of your neighborhood (air, water, soil, etc.)?
   2. Have you noticed any environmental health issues in your community that you believe are related to pet ownership?
2. How does the (natural or built) environment around you impact the health and wellbeing of your family and pets?
   1. For example, do you feel there is sufficient access to pet waste disposal facilities, such as designated bins or waste collection services?
3. Have you ever experienced any conflict or tensions with neighbors regarding pet-related environmental issues, such as noise or waste disposal?
4. Do you feel like you have adequate access to green spaces and natural areas (e.g., parks)?
   1. If not, do you feel like this has a negative impact on you/your family/your pets?
   2. How often do you take your pet to a green space or park for exercise?
